# Social media use by community-based organizations conducting health promotion: a content analysis

**DOI:** 10.1186/1471-2458-13-1129

**Published:** 2013-12-05

**Authors:** Shoba Ramanadhan, Samuel R Mendez, Megan Rao, Kasisomayajula Viswanath

**Affiliations:** 1 Center for Community-Based Research, Dana-Farber Cancer Institute, 450 Brookline Ave, LW 703, Boston, MA 02215, USA; 2 University of Michigan School of Public Health, 1415 Washington Heights, Ann Arbor, MI 48109-2029, USA; 3 Department of Social and Behavioral Sciences, Harvard School of Public Health, 677 Huntington Ave., Boston, MA 02115, USA

**Keywords:** Community-based organizations, Social media, Health promotion, Content analysis

## Abstract

**Background:**

Community-based organizations (CBOs) are critical channels for the delivery of health promotion programs. Much of their influence comes from the relationships they have with community members and other key stakeholders and they may be able to harness the power of social media tools to develop and maintain these relationships. There are limited data describing if and how CBOs are using social media. This study assesses the extent to which CBOs engaged in health promotion use popular social media channels, the types of content typically shared, and the extent to which the interactive aspects of social media tools are utilized.

**Methods:**

We assessed the social media presence and patterns of usage of CBOs engaged in health promotion in Boston, Lawrence, and Worcester, Massachusetts. We coded content on three popular channels: Facebook, Twitter, and YouTube. We used content analysis techniques to quantitatively summarize posts, tweets, and videos on these channels, respectively. For each organization, we coded all content put forth by the CBO on the three channels in a 30-day window. Two coders were trained and conducted the coding. Data were collected between November 2011 and January 2012.

**Results:**

A total of 166 organizations were included in our census. We found that 42% of organizations used at least one of the channels of interest. Across the three channels, organization promotion was the most common theme for content (66% of posts, 63% of tweets, and 93% of videos included this content). Most organizations updated Facebook and Twitter content at rates close to recommended frequencies. We found limited interaction/engagement with audience members.

**Conclusions:**

Much of the use of social media tools appeared to be uni-directional, a flow of information from the organization to the audience. By better leveraging opportunities for interaction and user engagement, these organizations can reap greater benefits from the non-trivial investment required to use social media well. Future research should assess links between use patterns and organizational characteristics, staff perspectives, and audience engagement.

## Background

Community-based organizations (CBOs) are essential, but often underutilized, channels for health promotion efforts [1,2]. Much of the impact of CBOs derives from their relationships with other CBOs, community members, and other stakeholders [3]. These relationships are the source of deep social capital, with rich resources embedded in social networks and structures that can be accessed and utilized to achieve goals [4]. This capital is promoted through ongoing engagement and communication with clients and organizations use a variety of tools for this purpose. Today, driven by information and communication technologies, social media are important tools, among others, for CBOs to engage clients and other stakeholders [5].

Social media tools and platforms reflect the shift away from static “Web 1.0” platforms towards “Web 2.0”: from unidirectional information flow to interactive, multi-directional communication capable of harnessing collective intelligence and user-generated content [6]. Examples of these tools include social networking sites, blogs, microblogs, wikis, and services to share multimedia content such as videos. Much of the power of these tools comes from engaging users as both creators and consumers of Internet-based content thus fostering and strengthening relationships between organizations and clients [7].

In the United States, social media use is growing rapidly. In 2012, online sources accounted for about one-third of media consumption [8] and social media channels accounted for almost one-quarter of time Americans spent online [9]. The use of social networking sites, such as Facebook and Twitter, increased among adult Internet users from 8% to 67% between 2005 and 2012 [10]. Much of this is driven by developments in information and communication technologies (ICTs), especially mobile media, such as tablets and cell phones [5,9]. Given these shifts, companies across the marketplace are using social media to build ties with customers, support their brands, and conduct market research [11-14].

Organizations in the public health arena have followed suit and are utilizing social media for health education, intervention, and social marketing efforts [15]. The Centers for Disease Control and Prevention (CDC) has recently begun to provide guidance for organizations to use these tools successfully [16]. In the health promotion arena, social media tools have a wide range of potential benefits including the abilities to disseminate information quickly, leverage community networks for information-sharing, reach broad audiences, customize health messages for specific groups, encourage interaction and engagement, and empower healthy decision-making [16,17]. Although social media represent tremendous opportunities for CBOs, they also reflect an important culture shift as they blur traditional boundaries between “experts” and the general public/consumers. Along with opportunities to harness collective wisdom, there are also challenges for organizations to maintain (or appropriately relinquish) control over content [7]. This requires a shift from the traditional command-and-control approach to a more participatory approach [5].

In addition to organizational challenges, social media and new technologies also carry a potential to create or exacerbate health disparities. For example, in 2011 in the US, factors such as older age (65+), lower household income (less than $20,000 per year), limited education (less than a high school degree), and limited English proficiency were linked to lower rates of Internet access [18]. This is consistent with the literature that finds that many of the social determinants typically associated with health inequalities (e.g. race, ethnicity, class, and geography) are also linked to communication inequalities [19]. By communication inequalities, we mean differences among social groups in the generation, manipulation, and distribution of information at the group level and differences in access to and ability to take advantage of information at the individual level [20]. We hypothesize that communication inequalities mediate the relationship between social determinants and outcomes, and thus serve as one explanation for health disparities [21]. We are thus prompted to study social media use among organizations that target the underserved to document communication inequalities and develop strategies for addressing them. At the same time, there is an important trend of increasing Internet access via mobile devices. For example, increased smartphone penetration is expected to narrow the “broadband gap” between African-Americans and Latinos versus whites [22], though this positive trend does not appear when stratifying by education or income levels [23]. Clearly, there is a complicated pattern of access among various underserved groups, but broad trends of increasing access support inquiry into these areas. This attention is particularly important given our group’s recent findings from a randomized controlled trial that provided home Internet and computers to low SES, novice users, accompanied by training on using these new tools. We found that once access issues are resolved, social media sites were a major focus of web activity for low SES households [24].

Despite the prominence of social media in discussions of CBOs’ efforts to engage the public and conduct health promotion, guidance on how best to use tools of social media remains limited. Guidelines for successfully integrating social media into a company’s broader communications plan and strategy are still being debated and developed [12]. Typically, organizations use new media for information-sharing and relationship-building [25]. Yet, recent reviews suggest that nonprofits typically use social media for administrative purposes, to educate the public about their services and offerings, and to share information with clients. These reviews highlight lost opportunities to leverage the interactive component of social media tools [26,27]. Given the potential impact of these relatively inexpensive tools, we sought to profile the use of social media among CBOs engaged in health promotion.

In this study, we focused on three research questions. First, to what extent are CBOs engaged in health promotion using popular social media channels? Second, what types of content do these CBOs typically share via social media? Third, to what extent do these organizations leverage the interactive nature of the social media tools? We assessed social media presence and patterns of usage among CBOs located in Boston, Lawrence, and Worcester, three diverse communities in Massachusetts.

## Methods

Data for this study come from PLANET MassCONECT, a knowledge translation project that builds capacity to adopt evidence-based health promotion programs among CBOs in Boston, Lawrence, and Worcester, Massachusetts [28]. The project utilizes a Community-Based Participatory Research (CBPR) approach that involves a range of stakeholders and partners in the research process to address questions of importance to the community and bring about mutual benefit through the integration of knowledge and action [29,30]. As part of the CBPR approach, we created a Community Project Advisory Committee (C-PAC), an advisory group of community partners, investigators, and study staff.

We were fundamentally interested in CBOs that engage in health promotion. We take a broad view of health promotion as the set of processes and actions that allow individuals, groups, and communities to engage with their health and improve it, through changes in a range of sectors and at a range of levels [31,32]. Given that lists of CBOs conducting health promotion are not available publicly, we created a roster of CBOs engaged in health promotion in Boston, Lawrence, and Worcester. We focused on non-profit organizations that are typically overseen by an elected board and engaged in work driven by the needs of community stakeholders [3]. We included organizations within the boundaries of Boston proper (as defined by the Boston Public Health Commission), the greater Lawrence community (including Methuen and North Andover), and the greater Worcester Area (including Leicester, Paxton, Holden, West Boylston, Boylston, Shrewsbury, Grafton, Millbury, and Auburn). These cities were chosen for the parent project because they represent tremendous diversity and offer a useful testbed for comparisons. Socio-demographic data for the three communities are presented in Table 1.

**Table 1:** **Socio-demographic profile of three study communities, 2007–2011 Census data**[33]

	**Population**	Race*					**Economics**	
		**White (%)**	**Black (%)**	**Asian (%)**	Other (%)**	**Hispanic or Latino (%)**	Median household Income ($)***	**Adults living below the poverty level (%)**
Boston	609,942	56.7	27.8	9.7	9.8	17.3	51,739	19.8
Greater Lawrence								
Lawrence	75,761	35.1	5.8	3.3	59.6	72.9	31,478	23.9
Methuen	46,785	80.7	2.8	5.1	13.9	17.6	65,799	7.6
North Andover	28,156	90.7	1.9	6.0	2.6	2.9	95,199	4.0
Greater Worcester								
Worcester	181,045	79.2	12.1	6.3	5.4	19.3	45,846	16.6
Leicester	10,934	96.5	1.4	2.0	0.5	4.1	72,471	5.3
Paxton	4,767	92.7	4.8	2.5	0.6	0.9	105,072	3.0
Holden	17,197	96.4	0.9	2.8	0.6	1.2	89,660	3.7
West Boylston	7,660	90.4	5.4	0.3	4.8	6.9	73,600	3.8
Boylston	4,320	97.6	0.0	2.0	7.4	3.0	91,734	1.2
Shrewsbury	35,269	81.1	2.1	17.3	2.0	2.4	88,985	4.1
Grafton	17,472	89.5	2.4	8.4	0.6	2.2	89,950	6.3
Millbury	13,250	96.2	0.7	2.9	1.0	3.0	77,883	2.5
Auburn	16,183	97.6	1.3	0.6	1.8	1.0	73,559	4.9

*Reported alone or in combination with other categories.

**Includes (alone or in combination): American Indian and Alaska Native, Native Hawaiian and Other Pacific Islander, and Some Other Race.

***In 2011 inflation-adjusted dollars.

Data describing Internet penetration for these cities were not available. As described above, we were prompted to assess potential communication inequalities at the organization-level as improving the ability of CBOs targeting the underserved to communicate effectively with constituents may be an important lever in the fight against disparities.

Our intention was to create a comprehensive list of CBOs engaged in health promotion in the three communities and we included organizations from the health sector as well as other sectors, such as education and housing. At the start of the PLANET MassCONECT project (2008), the study team started to compile the list by expanding a partner list from a previous CBPR project in these communities that included diverse organizations engaged in health promotion. The team then searched for additional organizations using the following websites (and search terms): 1) Google.com (city name + community + health organizations) and (city name + nonprofit + health), 2) Yellowpages.com (city name + nonprofit + health) and (city name + community + health), 3) IRS.gov, to obtain a list of 501(c)3 nonprofits in the three communities, and 4) the Attorney General’s website in each of the three communities. An important second step was to leverage the local knowledge of community health educators and C-PAC members in each of the three communities to expand the list as much as possible. We confirmed that the organization engaged in health promotion in any of the communities of interest to retain them on the list. While it is possible that some eligible organizations were left off the list, the closely-knit non-profit sectors in Lawrence and Worcester make this challenge less of an issue in those communities. In 2012, we updated that comprehensive list by redoing the web-based searches and engaging our community health educators to update the list. We excluded organizations that had closed or changed service area and added newly formed organizations. The final roster included 166 organizations.

Though social media can be described broadly, it is useful to consider the diversity of applications in this category. In addition to attracting users with different demographic profiles [34,35], social media tools also vary in terms of the types of interactions they support, the technical expertise required by consumers to engage and collaborate, and the intensity of engagement required, among other factors. In this study, we focused on Facebook, Twitter, and YouTube, which provide a broad, complementary set of popular exemplars. Our team conducted a preliminary scan of the use of social media sites by non-profits engaged in health promotion and found these three sites to be the most popular among CBOs in our target areas. A recent assessment of social media use by state health departments also supports this selection; they found that among departments using social media, 87% had a Twitter account, 56% had a Facebook account, and 43% had a YouTube channel. Other sites, such as Flickr, and other channels, such as blogs were not used to this extent [15]. As noted elsewhere [36], search strategies for social media are still being routinized and developed at this time.

Facebook is a social networking service that allows users to interact and share digital content in diverse ways, including sending messages, uploading photographs, and sharing comments on products on third-party websites [13]. In 2012, the site had over 152 million unique visitors from the United States and was the most popular social networking site on PCs and mobile web devices [9]. The company’s best practices for businesses stress: 1) real-time engagement with consumers; 2) utilization of interactive features; 3) extension of reach by targeting “friends” of existing clients and consumers; 4) use of an informal tone to engage in a new way with consumers, and 5) weekly posting at a minimum [37]. Guidelines for social media among health-focused organizations suggests daily posting and active monitoring of Facebook pages if there is a high level of activity [38].

Twitter supports social information-sharing through 140-character “tweets” or messages, which can be shared easily among users across a wide range of platforms and devices. In 2012, the service had about 37 million unique visitors [9]. For small businesses, the company provides similar suggestions to those offered by Facebook, with the exception of the promotion of applications. Twitter’s suggestions also emphasize the opportunity to: 1) demonstrate competence by linking to broader ideas in the field and 2) leverage the ability to share content (such as amplifying positive feedback by retweeting and replying publicly to positive tweets or sharing videos or other content that can be shared easily). The company suggests that businesses start by “tweeting” on a daily basis and adjusting as needed [39]. A recent study found that organizational activity (frequency of posting) was positively linked to consumer engagement and information-sharing. The authors noted that most retweets occur with 1.5 to 4 hours of the original, which hints at the lifecycle of a tweet [40].

YouTube is a video-sharing service that allows users to upload, view, and share videos via the Internet. Potential benefits for companies include the opportunity to engage emotionally with clients and customers through video. Companies can promote their videos internally and also can purchase advertising services to promote videos to viewers [41].

### Web presence assessment

As the first step in data collection, coders looked for a web presence for each CBO and then searched for Facebook, Twitter, and You Tube accounts. To gauge the appropriateness of our selection of channels, coders tracked links to other social media sites (e.g., Flickr, LinkedIn, and Google+) from the organization’s homepage.

### Content analysis methods - overview

Content analysis is an analytical technique that relies on the scientific method to quantitatively summarize messages [42]. The main concepts investigated in this project were: 1) the types of content shared by CBOs on key social media channels, 2) the use of interactive features of the social media sites, and 3) the response to content by audience members. We utilized human coding for this study.

### Units of data collection

For Facebook, the unit is each post generated by the organization. A “post” on Facebook was defined as an update of the channel with any of the following Facebook-specific avenues for sharing content: status updates, links, events, discussions, photos, notes, or videos. Items automatically added by Facebook summarizing the organization’s activity were not included. For Twitter, the unit of data collection is each tweet generated by the organization, whether original content or a rebroadcasting of content from another account. For YouTube, the unit is a single video posted by the organization.

### Codebook and coding form

A 27-page codebook was developed to define the following measured variables for each unit of data collection. The coding scheme was developed based on the literature and an environmental scan of the Facebook and Twitter accounts of 50 local, regional, and national non-profits engaged in health promotion. We used an iterative process of pilot-testing and refining the codebook. We then trained the two coders on the codebook and conducted an inter-coder reliability test on approximately 20% of the content in the study. For all variables with inter-coder reliability below the accepted threshold (0.70) [43], we resolved all points of disagreement through consensus coding. Resource limitations for the pilot study prevented additional tests.

Each unit linked to the source organization and date of posting. Then, each unit was coded for presence or absence of content in a series of categories, presented in Table 2. Thus, a single post, tweet, or video could be coded as having multiple categories of content (e.g. a post could contain content related to organization promotion and cross-promotion).

**Table 2:** Classification scheme for Facebook, Twitter, and YouTube content

**Category of content**	**Definition**
Fundraising	Content that serves as a solicitation, e.g. advertising merchandise, soliciting donations, or selling tickets to a fundraising event.
Health education/news	Educational information or news articles on a range of health topics, e.g. health tips, policy decisions that relate to health, and scientific findings.
Human interest	Content that tells a personal story about a given health topic or public health initiative.
Material for professionals	Content that is targeted at health professionals, including job postings and professional development.
Miscellaneous	Content which does not fit into any of the other categories.
Non-informational	Content that is meant to maintain connections, but serves no informational, promotional, or persuasive purpose, e.g. holiday greetings or inspirational quotes.
Organization promotion	Content that advertises or builds the image of the organization sponsoring the account. e.g. organization-specific news, event/program updates, service offerings, and summaries of past events.
Cross-promotion	Content that advertises or builds the image of another organization, e.g. news or events.

In addition to thematic content, coders assessed presence of links to internal content (content held by the source organization), external content (content held by other organizations). For Facebook, the presence of links to both types of content was recorded. For Twitter, coders counted the number of hashtags used within each tweet. The hashtag (#) is a symbol used to identify the theme of the tweet so that users can click on a hashtag to find similarly-themed tweets or find a given tweet in a search [39]. Coders also counted the number of Retweets, or tweets that were taken from another Twitter user’s account and shared with the account holder’s audience [39]. Last, coders assessed high-level features of audience engagement for two of the channels. For Facebook, coders assessed the number of “likes” each unit generated. “Likes” represent user engagement and accumulate when the audience presses the “like” button, a feature available for each individual post and its comments. For YouTube, coders tracked the number of comments each unit generated.

In addition to analyzing posts, we also collected high-level data about engagement. For Facebook, coders recorded the number of “likes” or “friends” an organization had. Organizations can create a Facebook “page” which can be “liked” by Facebook users. Some organizations still maintained pages that were set up for individuals, and thus had “friends” the way an individual user might. Typically, companies, brands, and products are represented by pages that allow Facebook users to express their interest and approval by liking them. When a user likes a page, that page’s posts will regularly appear on the user’s Newsfeed alongside the posts of the user’s friends and other liked pages. Accordingly, a Facebook user can not only like a page, he or she can also like individual posts generated by the page owner, which is yet another way for the Facebook user to endorse his or her preferences. For Twitter, a high-level metric of importance was the number of “followers,” or the number of users who have subscribed to receive tweets from the organization. For YouTube, the high-level metric was the number of “subscribers,” the number of users who have subscribed to receive updates about the organization’s content. These metrics reflect publicly accessible data.

### Sample

For each organization on the roster, the universe of the content is defined as follows: 1) a census of Facebook posts generated by the organization in the 30 days prior to coding start, 2) a census of Twitter tweets generated by the organization in the 30 days prior to coding start, and 3) the 5 most recently posted YouTube videos. Given that we were interested in linked content, we were unable to use screenshots to capture all of the data for all CBOs on a single day. Thus, we conducted the data collection for each organization over a 2-day period and assessed the prior 30 days of activity from data collection start. Organizations were randomly ordered and data were collected between November 2011 to January 2012.

### Data analysis

Much of the analysis was descriptive and presents counts from the coding process. To assess the distribution of content across key categories on Facebook, we created a summary statistic for each account that described the frequency with which a given theme appeared in posts. Then, these summary statistics were averaged to create the summary data presented. We presented the data focused on organizations rather than individual posts to be consistent with our research focus at the organization level. The same processes were used for Twitter and YouTube content. After the quantitative analysis was completed, data were presented to members of our C-PAC to discuss and refine the study team’s preliminary interpretations of the data.

## Results

We surveyed social media usage among 166 CBOs engaged in health promotion in the three communities. We found that 162 of these (98%) had a website. A total of 70 organizations (42%) had a social media presence on Facebook, Twitter, and/or YouTube. The CBOs that did not have a presence on the three sites of interest did not advertise a presence on other social media sites. Among CBOs using one of the three channels, we found that 18 organizations had a presence on one site beyond our focus and 3 organizations had a presence on two sites beyond Facebook, Twitter, and YouTube. The majority of the organizations using social media (81%) highlighted their use of these tools on their website’s homepage. A total of 69 organizations had a Facebook account (42% of total), about a quarter (24%) had a Twitter account, and 13% had a YouTube account. Of note, some organizational subunits of a national organization linked to Twitter and YouTube accounts run by a higher-level unit of that organization. These subunits were treated as distinct organizations for the purposes of the analysis because they operate independently at the local level.

### Facebook

Although 42% of CBOs in the study had Facebook accounts, not all were active on this channel during our 30-day data collection windows. The 60 organizations that were active (36% of all CBOs in the study) had an average of 543 friends or likes, depending on the type of account they used. They generated a total of 898 posts in the 30-day period, with an average of 14.97 posts (SD = 13.06). The median number was 11, with a minimum of 1 and a maximum of 53. When compared to published guidelines from Facebook (which suggest weekly posting at a minimum), we found that 23 organizations (38% of organizations with activity in the last 30 days) had at least one period of eight or more days from their last post. These organizations had an average of 1.26 gaps of 2 or more days in the 30-day data collection period. Compared to the more stringent suggestion of daily posting, almost all (97%) had at least one period of two or more days from their last post. These organizations had an average of 4.45 gaps of 2 or more days in the 30-day data collection period.

To understand the ways in which organizations were utilizing Facebook posts, we looked at the distribution of post categories within each account. As seen in Table 3, we found that the top three types of content were: organization promotion (66%), health education/news (24%), and cross-promotion (19%).

**Table 3:** Average distribution of content in coded categories in Facebook posts (n = 60 accounts/898 posts), Twitter tweets (n = 36 accounts/965 posts), and YouTube videos (n = 18 accounts/86 videos)

Category *	**Presence in Facebook posts (%)**	**Presence in Twitter tweets (%)**	**Presence in YouTube videos (%)**
Organization promotion	66	63	93
Health education/news	24	25	12
Cross-promotion	19	20	0
Fundraising	12	12	6
Non-informational	6	6	0
Miscellaneous	5	9	0
Human interest	2	2	31
Material for professionals	0	0	0

*multiple selections permitted.

We also looked at ways in which CBOs leveraged the multimedia and relational aspects of the Facebook platform. About one-third (32%) of total posts included links to external content; a slightly smaller percentage (27%) included links to internal content. Less than one-quarter (22%) of posts included a photo and less than 1% of posts included a video. Finally, we assessed audience engagement with the posts via the “like” function. Almost two-thirds (65%) of posts received at least one “like.” Stories with a human interest component, though a small proportion of overall posts, received the highest average number of “likes” per post, with an average of almost 6 “likes” per post. Additional details are provided in Table 4.

**Table 4:** Average number of “likes” per Facebook post, by category, in decreasing order

	**Number of posts in category**	**Mean**	**Standard deviation**	**Min**	**Max**
Human interest	20	5.85	8.98	0	38
Non-informational	67	3.73	5.82	0	38
Organization promotion	622	2.46	3.67	0	36
Cross-promotion	146	2.44	5.97	0	64
Miscellaneous	44	2.25	3.26	0	18
Health education/news	213	2.06	5.05	0	64
Fundraising	89	1.37	1.82	0	12
Material for professionals	3	1.33	1.15	0	2

### Twitter

A total of 40 CBOs in our study (about 24%) had a Twitter account. As noted above, some organizations used the Twitter account of a higher-level unit of that organization. Of the 37 unique Twitter accounts, 36 were active during the study window and had an average of 695 followers. The 36 active accounts generated 965 tweets for coding. The average number of tweets per organization over a 30-day period was 26.81 (SD = 27.87). The median number of tweets was 17.5, with a minimum of 1 and a maximum of 100. When compared to Twitter guidelines suggesting daily tweets, almost all organizations (92%) had a period of at least two days in which the organization did not tweet. These organizations averaged 4.06 gaps of at least two days in the 30-day data collection period. We next looked at the distribution of subject categories of tweets within each account. The top three types of content were: organization promotion (63%), health education/news (25%), and cross-promotion (20%). Additional details regarding other categories are provided in Table 2.

To complement the analysis of the content of the tweets, we also assessed ways in which CBOs leveraged the relational aspects of the Twitter platform. About one-third (34%) of posts linked to the organization’s own content and a similar percentage (32%) linked to external content. An important aspect of Twitter is the ability to share tweets from one account to another, thus enabling the spread of information. We found that 21% of all tweets were retweets (16% were retweeted from an organization and 5% were retweeted from an individual). About 38% included a mention (31% included a mention of an organization and 11% included a mention of an individual). About 37% of all tweets included a hashtag (#).

### YouTube

A total of 21 organizations (13%) in the study had a YouTube account. As with the Twitter accounts, some CBOs linked to the YouTube account of their umbrella organizations. A total of 19 organizations linked to accounts active in our 30-day window; 18 of these were unique. The average number of followers per account was 15.95. The 18 unique accounts generated 86 videos for coding, with an average of 4.78 videos (SD = 0.73). The median number of videos was 5, with a minimum of 2 and a maximum of 5. We found that, on average, organization promotion was the most common theme identified (93%). The second and third most frequent subject categories were human interest (31%) and health news/education (12%). The videos generated an average of 148.91 views (SD = 207.00), with a range of 1 to 1,231 views. Only 10% of videos had viewer comments.

## Discussion

Our study focused on the extent to which and ways in which CBOs utilize popular social media channels. We found that less than half of the CBOs in the study (42%) were using social media. Our results highlight opportunities for CBOs to better leverage the interactive features of social media as part of a strategic communication plan focused on engaging end-users.

Among CBOs utilizing social media, we found that many were using the tools in a manner that is typical of Web 1.0, with an emphasis on “pushing” information to users, rather than encouraging participation and engagement. For example, organization promotion was the dominant content type across posts, tweets, and videos in the study. At the same time, the usage of interactive features on social media sites was low, though organizations appeared to be taking greater advantage of the features available on Twitter. These results parallel recent findings from studies of state health departments [15], other nonprofits [26,27], health promotion programs targeting sexual health [36], and advocacy groups [44]. This highlights an important missed opportunity as user engagement can provide opportunities to develop and strengthen relationships, develop group identity, harness community intelligence, and motivate action [13,45,46].

One way to improve engagement is to provide offerings beyond advertising content, such as expert information [39,47,48]. For CBOs, this content may relate to health education/news, which was only included in about one-quarter of the content for Facebook and Twitter accounts. Another opportunity may lie with the “human interest” pieces, which had the greatest number of “likes” per post among Facebook posts. The small number of posts and wide distribution of “likes” limits the impact of the current finding, but may point to the power of narratives for engaging audiences and distinguishing the organization from others that may have similar offerings [12]. This is also consistent with a study of corporate tweets, which found that tweets are more likely to be shared if they elicit emotions (often containing humorous, political, or philanthropic themes) [40]. Solicitation of user-generated content, such as prompting users to take polls, upload videos or photos, etc. are low-cost, strategic techniques that can increase engagement.

In the context of strategic communications, CBOs need to think critically about whether, which, and how many social media tools to use. Coders detected much repetition of content between Facebook and Twitter, which can be effective if the audiences are distinct, but otherwise may be redundant. Assessing both the demographic profiles of various channels, as well as the penetration of channels among the target audience will allow CBOs to strategically target communications. Important differences among social media sites in terms of age, gender, race/ethnicity, education levels, and rural–urban location of users have been documented [34,35]. Social media-related decisions will also be influenced by resources requirements given that effective use of social media tools requires regular content contributions and active monitoring [11]. Given the resource constraints that CBOs may face (particularly local organizations like many of the organizations in this study), it is important that the decision to utilize these tools be made carefully as outdated and unmonitored social media outlets can harm the reputation of the brand.

The results of the study also point to themes that may be useful in future assessments. Further investigation into the details of organization promotion is warranted as this category is quite broad. Also, given the interest in relationship-building as an important leverage point for CBOs, it may also be useful to delve more actively into sub-categories of content that support relationships. One example is content aimed at eliciting responses, such as questions posed to the community [25]. On the other hand, categories such as professional development, came up in our preliminary coding, but were rarely used during data collection.

As noted earlier, as CBOs enter the social media space, it is important to consider potential communication inequalities. Social media are emerging as important tools for communication with the advantage of generating consumer engagement. If an organization does not exploit this new tool effectively, it is likely to be at a disadvantage; this has the potential to widen inequalities among organizations. At the individual level, the traditional communication inequalities are muted when it comes to social media use, providing great potential for bridging access to health information [49]. While the use of social media for health interventions was beyond the scope of this study, such complementary activities may also be supported by CBO engagement with communities.

There are some limitations that help place the results in context. First, this study looked at one subsection of social media use – the content put forth by CBOs. Data describing audience usage as well as CBO organizational characteristics and perspectives on the use of social media will be needed to provide a full picture. Collection of this data was beyond the scope of this pilot study, but the detailed description of CBO use of channel features can inform future work. As seen in a study of large nonprofits, strategy, organizational capacity, governance structures, and environmental factors all may play a role in the use of social media tools [50]. Second, the communities under study were not selected randomly. However, these communities were selected for the initial project as they represent a diverse range of population and environmental characteristics. Third, there are no publicly available lists of CBOs conducting health promotion locally, so we may have missed some eligible organizations. However, we used a range of search strategies and leveraged local knowledge to develop as comprehensive a list as possible. Finally, our data were collected between November and January and there may have been variation in content and frequency of posting based on the timing of the data collection in relation to holidays. We attempted to mitigate this challenge by assigning the data collection schedule randomly and using a 30-day window for data collection. Despite these limitations, the study has a series of important strengths. First, we are unaware of published studies describing patterns of social media use by CBOs engaged in health promotion and the study provides useful preliminary data. By understanding patterns within local organizations, we can identify opportunities and strategies that fit available resources for local organizations. Second, by including organizations that engage in health promotion, regardless of their overall mission, we have captured a broad range of CBOs that may contribute to health promotion in community settings. Finally, we focused on a broad, complementary set of social media exemplars that were popular among the organizations in our study. Future research should examine the relationships between organizational characteristics and social media use (with an emphasis on the motivations and perspectives of CBO leaders and staff) as well as the needs and preferences of the intended target audience.

## Conclusions

As with any new tools, there are not only tremendous opportunities, but a great deal of debate regarding how best to use social media to engage with audiences. By taking better advantage of the interactive and engagement-oriented features of these tools, CBOs can utilize social media as an important complement to existing communication efforts. Given that social media use is growing rapidly and across diverse population groups, it is an ideal time for CBOs engaged in health promotion to consider the investment as part of their broader strategic communications plan. By developing new relationships and strengthening existing connections, CBOs may be able to use social media to increase their impact on the health of the communities they serve.

## Competing interests

The authors declare that they have no competing interests.

## Authors’ contributions

SR and KV conceived of and designed the study. SR, SM, and KV designed the codebook. SM and MR collected the data. SR analyzed the data. All authors engaged in data interpretation and manuscript development. All authors read and approved the final manuscript.

## Authors’ information

SR is a Research Scientist at the Center for Community-based Research at the Dana-Farber Cancer Institute. SM is an undergraduate at Harvard University. MR is a graduate student at the University of Michigan School of Public Health and was an intern at Dana-Farber during the data collection phase of this study. KV is a Professor at the Center for Community-based Research at the Dana-Farber Cancer Institute and the Department of Social and Behavioral Sciences at the Harvard School of Public Health.

## Pre-publication history

The pre-publication history for this paper can be accessed here:

http://www.biomedcentral.com/1471-2458/13/1129/prepub
